# Efficacy of Osteoporosis Medications in Patients with Type 2 Diabetes

**DOI:** 10.1007/s11914-023-00833-3

**Published:** 2023-12-14

**Authors:** Tatiane Vilaca, Richard Eastell

**Affiliations:** 1 Mellanby Centre for Musculoskeletal Research, Division of Clinical Medicine, University of Sheffield, Sheffield, UK; 2 Present Address: Metabolic Bone Centre – Northern General Hospital, Herries Road, Sheffield, S5 7AU UK

**Keywords:** Type 2 diabetes, Osteoporosis, Fractures, Anti-osteoporosis treatment

## Abstract

**Purpose of the Review:**

The purpose of the review is to summarise the current scientific evidence on the efficacy of osteoporosis medications in patients with type 2 diabetes.

**Recent Findings:**

Type 2 diabetes (T2D) is a growing global epidemic. The highest prevalence is observed in the elderly, the same population affected by osteoporosis. Despite normal or even increased bone mineral density and low bone turnover, T2D is associated with an increased risk of fractures in most skeletal sites. These findings raised concerns over the efficacy of anti-osteoporosis drugs in this population. There is no randomised controlled trial designed specifically for people with T2D. However, observational studies and post-hoc analyses of randomised controlled trials have provided valuable insights into the effects of various anti-osteoporosis treatments in this population. Overall, most anti-osteoporosis drugs seem to have similar efficacy and safety profiles for people with and without type 2 diabetes. However, continued research and long-term safety data are needed to optimise treatment strategies and improve bone health outcomes in this population.

**Summary:**

The current evidence suggests that most anti-osteoporosis drugs exhibit comparable efficacy in people with and without T2D.

## Introduction

Type 2 diabetes (T2D) has reached epidemic proportions worldwide, with an alarming increase in its prevalence over the past few decades. According to the International Diabetes Federation, an estimated 463 million adults were living with diabetes worldwide in 2019, and this number is projected to rise to 700 million by 2045 [1]. Population-specific incidence rates vary depending on demographic, genetic, and lifestyle factors. Age is a significant risk factor. Despite the rising prevalence of T2D among younger individuals, often attributed to sedentary lifestyles and poor dietary habits, older adults still show a higher prevalence [1]. This is the same population most affected by osteoporosis.

Osteoporosis is characterised by low bone mass and deteriorating bone microarchitecture. Both modifiable and non-modifiable risk factors contribute to the development of the disease. Non-modifiable risk factors include age, gender, and family history of fractures or osteoporosis. Female gender plays a critical role due to the rapid decline in oestrogen levels after menopause, which leads to accelerated bone loss. Modifiable risk factors include inadequate calcium and vitamin D intake, a sedentary lifestyle, smoking, excessive alcohol consumption, and long-term use of certain medications, such as glucocorticoids and aromatase inhibitors [2].

The incidence of osteoporotic fractures increases with age, making osteoporosis a significant contributor to the global burden of musculoskeletal diseases. Osteoporosis-related fractures, particularly hip fractures, have significant consequences for individuals and healthcare systems, leading to impairments in mobility, increased healthcare costs, and decreased quality of life. Hip fractures are particularly associated with significant morbidity and mortality rates, impairing mobility and independence among affected individuals. The resulting hospitalisations and long-term care requirements impose substantial economic burdens on healthcare systems. Moreover, the fear of falling and decreased quality of life are common psychosocial impacts experienced by individuals living with osteoporosis [2]. As the global population ages, the burden of osteoporosis and its associated fractures is expected to rise substantially.

In this narrative review, we summarise the current scientific evidence on the efficacy of osteoporosis medications in patients with T2D. We searched PubMed using a combination of terms, including ‘osteoporosis treatment’, the drugs’ classes and names, and ‘diabetes’. We also searched for key papers in the field and their references. Following an initial screening based on titles and abstracts, relevant articles were thoroughly examined in full text. We included articles written in English and pertinent to the scope of our review.

## Bone Fragility in T2D

T2D is linked to an increased risk of fractures. Overall, those with T2D had a higher risk of hip [3–5], upper arm [4], ankle [4, 6], and non-vertebral fractures [3] than people without diabetes. This increase in the risk of fractures is observed despite the high body mass index (BMI) often observed in T2D. A high BMI is usually associated with a lower risk of hip fractures [7]. This paradoxical finding highlights the unique scenario observed in T2D. The increase in risk of fractures varies from 15 to 54%, namely 15% for ankle fractures, 19% for non-vertebral fractures, 22% for any fracture, 33% for hip fractures, and 54% for upper arm fractures. Younger people, those using insulin, and those with diabetes for a longer period had a higher risk of fractures in T2D [3]. A recent study has investigated the trends in vertebral, hip, humerus, forearm, foot, or ankle fracture rates among patients with and without T2D for 21 years, up to 2017, in Denmark. The study found fracture rates were higher in patients with T2D than those without diabetes, except for foot fractures. However, fracture rates decreased over time for both groups at all sites except for vertebral fractures, which increased in people with and without T2D [8]. This increase in vertebral fractures is probably associated with greater detection of these fractures since most of them are often not diagnosed. Interestingly, when adjusted for age, differences in hip fracture rates between patients with and without T2D were not observed after 2004, and similar findings were reported for humerus fractures after 2010 [8]. These findings suggest that more recent advances in managing T2D may impact the fracture risk in this population, but the mechanism is unknown.

The increase in the risk of fractures in T2D is independent of the presence of low mineral density detected by DXA. A meta-analysis has reported an increase in the risk of hip fracture and a paradoxical increase in bone mineral density in T2D [5]. A recent study has shown higher areal mineral bone density and favourable microarchitecture in individuals with T2D compared to controls [9]. These findings raise concerns regarding using BMD as a predictor of fractures in T2D. Longitudinal studies have shown that BMD predicts fractures in T2D; however, the risk of fractures for the same bone mineral density is higher in men and women with T2D compared to people without T2D [10]. Similarly, fracture risk prediction tools such as FRAX underestimate the risk of fractures in people with T2D [10].

## Diagnosis and Management of Osteoporosis in T2D

**Fig. 1:**
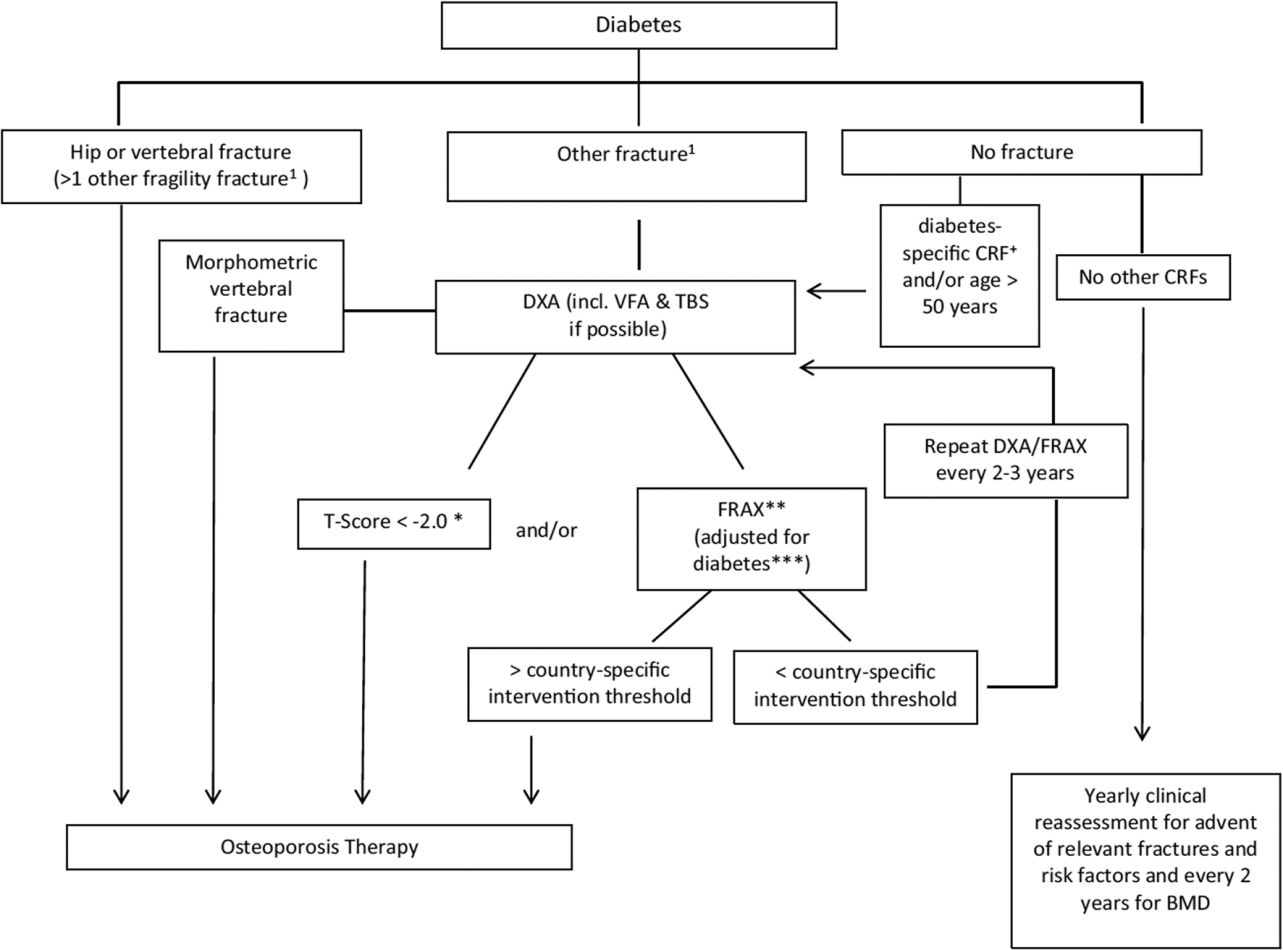
Fracture risk evaluation in patients with diabetes. *In diabetes, fracture risk at T-score <  − 2 equivalent for non-diabetes at T-score <  − 2.5 (see text). **Depending on country-specific guidelines for therapies. ***For example, with TBS and/or ‘RA’ – yes. + Diabetes-specific CRFs are listed in the text. Reproduced with permission from Ferrari et al. [11]

The diagnosis of osteoporosis in T2D follows the criteria applied to the general population based on low BMD and the occurrence of fragility fractures [2]. Due to the particularities of bone fragility in T2D, diagnosing and managing osteoporosis in this population is challenging. The Bone and Diabetes Working Group from the International Osteoporosis Foundation (IOF) has proposed an algorithm for the management of these patients (Fig. 1) [11]. Because, for a given fracture risk, people with T2D have a BMD 0.5 T-score higher than people without T2D [10], treatment is recommended for people with T2D with a T-score equal to or lower than 2.0. The common risk factors for osteoporosis, such as old age, female sex, low body mass index, previous fracture, family history of hip fracture, current smoking, rheumatoid arthritis, the use of glucocorticoids, recurrent falls, and low BMD, are also important in the population with T2D. However, diabetes-specific risk factors such as longer disease duration (greater than 5 years), use of certain medications such as insulin and thiazolidinediones, poor metabolic control (HbA1c > 7%; 53 mmol/mol), and the presence of microvascular complications (neuropathy, retinopathy, or nephropathy) should also be assessed [11]. Noteworthy, T2D might compromise vision, proprioception, and balance, leading to an increased risk of falls.

## Efficacy of Anti-Osteoporosis Medication in T2D

Observational studies and post-hoc analyses of previous randomised controlled trials (RCT) provide the current evidence on the efficacy of anti-osteoporosis drugs in patients with T2D since there are no randomised controlled trials (RCT) in this population.

### Anti-Resorptive Drugs

#### Bisphosphonates

Bisphosphonates (BP) are synthetic analogues of pyrophosphate. They selectively bind to bone mineral, particularly hydroxyapatite crystals, and are taken up by osteoclasts, suppressing bone resorption. This reduces the bone turnover rate and shifts the balance between bone formation and resorption, increasing BMD and skeleton strength. There are several types of BP, including nitrogen-containing BP, such as alendronate, risedronate, zoledronate, and ibandronate, and non-nitrogen-containing BP, such as etidronate. Alendronate and risedronate are oral medications prescribed weekly for osteoporosis treatment, while zoledronate is a potent intravenous BP prescribed yearly. Ibandronate is available in oral (monthly) and intravenous (every 3 months) formulations. Etidronate, a non-nitrogen-containing bisphosphonate, is less potent and is currently less used. BPs are widely used in the treatment of osteoporosis, as they reduce the risk of fractures [12].

The safety profile of BPs has been the subject of considerable research and clinical scrutiny. The most common side effect of oral BP is gastric discomfort, usually prevented by recommendations such as maintaining an upright position after oral administration. An acute-phase reaction is common after intravenous zoledronate. This is a systemic inflammatory response triggered by the release of pro-inflammatory cytokines, leading to fever, flu-like symptoms, myalgia, arthralgia, and mild leucocytosis. The activation of innate immune cells primarily drives the reaction, and management involves treatment with analgesics, antipyretics, and hydration [13].

One notable safety concern associated with BP therapy is the occurrence of atypical femur fractures. These fractures are uncommon but have been reported in some individuals, particularly those on long-term BP treatment. The fractures display unique patterns, different from typical osteoporotic fractures, and may occur with minimal or no trauma. However, it is essential to note that the absolute risk of atypical femur fractures remains low compared to the significant benefits of BPs in reducing overall fracture risk [14]. Another infrequent but serious side effect linked to BP use is osteonecrosis of the jaw (ONJ). ONJ is characterised by the exposure of jawbone tissue, leading to pain, infection, and delayed healing after dental procedures or trauma. The risk of ONJ is higher in patients receiving high-dose intravenous BP for cancer treatment, and it remains relatively rare in those receiving BP for osteoporosis. T2D has been associated with low bone turnover, which raised concerns about the adverse effects of anti-resorptive drugs in this population. However, evidence shows that these adverse events are not more common in people with T2D [15, 16].

BPs are effective in the treatment of osteoporosis in patients with T2D. Data from a nationwide cohort in Denmark compared people who had been given BP to people of the same age and gender in the general population. They found that the effectiveness of the anti-fracture treatment was the same for people with and without T2D. This study included people using alendronate, risedronate, and etidronate [17]. Post-hoc analysis from the Fracture Intervention Trial (FIT) showed that alendronate treatment increased BMD at all sites studied in women with T2D. Alendronate was well tolerated and considered an effective treatment for osteoporosis in women with T2D [18]. A post-hoc analysis of three Japanese trials with both men and women showed that T2D did not affect the response to risedronate treatment regarding BMD, bone turnover markers, or safety [19].

The most robust evidence for the efficacy of anti-resorptive therapies comes from a pooled analysis of individual participant data of RCT of these medications. The data was gathered for the Foundation for National Institutes of Health-American Society for Bone and Mineral Research-Study to Advance Bone Mineral Density as a Regulatory Endpoint, the SABRE cohort [20]. This is a unique dataset of individual patient data from randomised, placebo-controlled trials of osteoporosis therapies. The nine BP trials were grouped in a subgroup analysis. The authors used Cox regression to calculate the treatment-related hazard ratio (HR) for incident non-vertebral, hip, and all fractures and logistic regression to calculate the treatment-related odds ratio (OR) for incident radiographic vertebral fractures, separately for T2D and no diabetes. Using linear regression, the effect of treatment on the 2-year change in BMD by diabetes status was estimated. They evaluated the interaction between treatment and diabetes status in every study. No interaction between the treatment effect and diabetes status was observed in any of the nine trials of BP nor in the pooled analysis for these drugs (Table 1). The effect of BP on BMD and bone turnover markers was similar in the population with and without T2D [20]. This data shows that BP effectively reduces fracture risk and increases bone density, regardless of diabetes status.

**Table 1:** Comparison of the effect of treatment on changes in bone mineral density (BMD) and bone turnover markers (BTM) in people with and without T2D in nine bisphosphonate trials

	Non-DM		T2D		Interaction *p*^a^
	*N*	Mean (95% CI)	*N*	Mean (95% CI)	
% Difference in BMD change (active-placebo) at 24 months					
Total hip	17,612	3.66 (3.53–3.78)	716	4.08 (3.42–4.75)	0.20
Femoral neck	19,869	2.89 (2.74–3.04)	791	3.13 (2.27–3.99)	0.57
Lumbar spine	13,054	4.33 (4.17–4.49)	385	4.62 (3.76–5.47)	0.46
% Difference in BTM change at 3 to 12 months (active-placebo)					
CTX	5789	− 52.1 (− 53.7, − 50.5)	197	− 51.1 (− 59.8, − 40.5)	0.88
P1NP	7094	− 50.3 (− 51.4, − 49.2)	265	− 44.2 (− 50.6, − 36.9)	0.09
NTX/Cr	3774	− 37.6 (− 39.8, − 35.2)	119	− 41.5 (− 51.5, − 29.3)	0.53

All results are adjusted for trial

*BMD* bone mineral density, *BTM* bone turnover marker, *CTX* serum C-terminal cross-linking telopeptide, *NTX/Cr* urinary N-telopeptide of type I collagen/creatinine, *P1NP* serum procollagen type I N-propeptide

^a^2-way interaction: Treatment × Diabetes status

Reproduced with permission from Eastell et al. [20]

#### Denosumab

Denosumab is a monoclonal antibody that inhibits the receptor activator of nuclear factor-kappa B ligand (RANKL), a critical regulator of bone resorption. By binding to RANKL, denosumab effectively blocks its interaction with the RANK receptor, reducing osteoclast differentiation, activity, and subsequent bone resorption. This increases BMD and improves bone strength.

While denosumab has demonstrated efficacy in preventing fractures and increasing BMD, its use is associated with potential side effects. Some of the most common side effects of denosumab are skin reactions, and long-term use has been linked to an increased risk of ONJ and atypical femur fractures. Another important consideration with denosumab is the ‘bone turnover overshoot’ phenomenon upon discontinuation. After more than 2 years of denosumab use, discontinuation leads to increased bone resorption and an elevated risk of multiple vertebral fractures. To address this concern, denosumab discontinuation should be followed by BP use; however, the ideal schema remains to be defined [21, 22].

In a post-hoc analysis of the subgroup with T2D of the Fracture Reduction Evaluation of Denosumab in Osteoporosis every 6 Months (FREEDOM) study and its long-term extension, denosumab substantially increased BMD and decreased the risk of vertebral fractures in women with osteoporosis and T2D; however, the incidence of non-vertebral fractures was higher with denosumab than with placebo in subjects with T2D (11.7 vs. 5.9% *p* = 0.046) [23]. This finding was mainly driven by the observation of rib and ulna fractures only in patients with T2D receiving denosumab, while more hip fractures were observed in the group with T2D receiving placebo (*n* = 4) than the group receiving denosumab (*n* = 1), but this difference was not statistically significant (*p* = 0.145) [23]. Moreover, in the SABRE study individual trial analysis, participants with T2D showed an increased risk of non-vertebral fracture, and there was a significant interaction between the effects of denosumab and T2D for the non-vertebral fracture risk analysis [20]. Therefore, denosumab was effective in reducing vertebral fractures in people with T2D. There was no difference in the analysis for hip fracture, but there was an increase in the risk of non-vertebral fractures in women with T2D with the use of denosumab.

#### SERMs

Selective oestrogen receptor modulators (SERMs) are synthetic compounds that interact selectively with oestrogen receptors, exhibiting estrogenic effects on bone tissue while displaying antiestrogenic properties in other tissues like the breast and uterus. This unique mode of action provides a promising approach to addressing bone loss associated with oestrogen deficiency, especially in postmenopausal women, without some undesirable side effects observed with traditional hormone replacement therapy [24]. SERMs bind to oestrogen receptors within bone cells and increase osteoblast activity and bone formation. This results in an overall positive impact on BMD and bone strength. Moreover, SERMs simultaneously inhibit osteoclast-mediated bone resorption, further contributing to their beneficial effects on bone health. However, SERMs may be associated with certain side effects, such as hot flashes and venous thromboembolism [24].

Among the most widely studied SERMs for osteoporosis treatment is raloxifene. Raloxifene has been shown to reduce the risk of vertebral fractures and improve BMD in postmenopausal women with osteoporosis. It also protects against oestrogen receptor-positive breast cancer, making it an attractive option for women at risk for osteoporosis and breast cancer. Data from the Raloxifene Use for The Heart (RUTH) trial showed that treatment with raloxifene for 5 years reduced the risk of clinical vertebral fractures in postmenopausal women, regardless of the presence of T2D. Noteworthy, there was no reduction in the risk of non-vertebral fractures, including hip, femur, and wrist fractures [25]. In the Multiple Outcomes of Raloxifene Evaluation (MORE) study, raloxifene was more effective at preventing vertebral fractures in women with T2D than in those without T2D [26]. However, raloxifene is not commonly used due the lack of efficacy against non-vertebral fractures and the concern that there may be increased stroke risk, the latter being of importance in patients with diabetes. Nevertheless, the current evidence suggests similar efficacy of SERMs in women with and without T2D.

#### Overall Efficacy of Anti-Resorptive Drugs

**Fig. 2:**
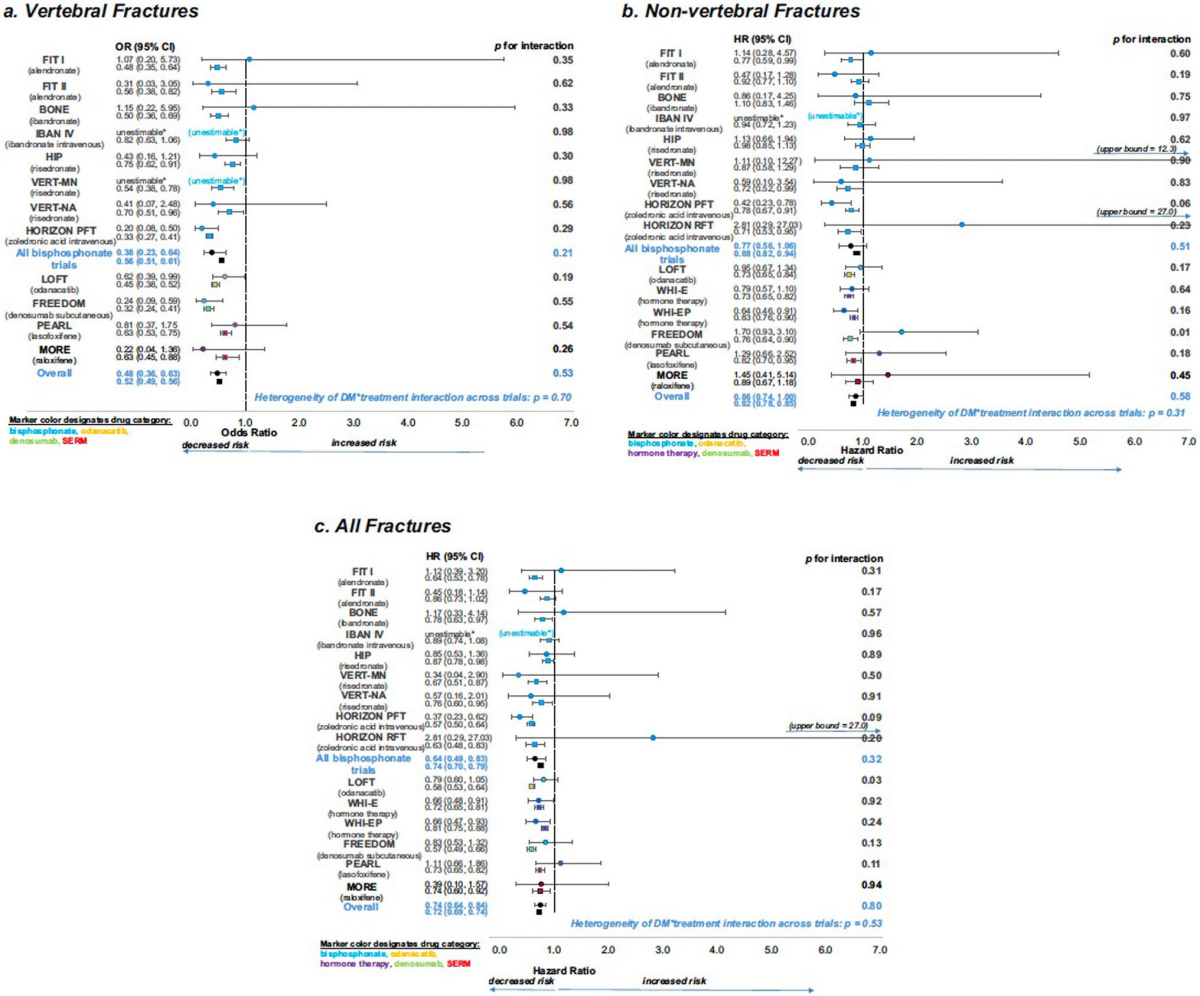
Forest plots showing the effects of treatment on fracture risk in T2D (solid circle) and non-DM (solid square). **a** Vertebral fractures, **b** non-vertebral fractures, and **c** all fractures. The *p* values for T2D status × treatment interaction for each trial, the overall effects, and the *p* value for heterogeneity of T2D status × treatment interaction across trials are all shown. *unestimable: no fractures in active and/or placebo groups in T2D. Reproduced with permission from Eastell et al. [20]

The SABRE study offers compelling evidence that the presence of T2D has no bearing on the effectiveness of antiresorptive treatments for osteoporosis, as both people with and without T2D experienced similar increases in bone density and decreases in fractures. The study analysed data from 15 randomised trials of osteoporosis therapies, including BP, SERMs, menopausal hormone therapy, denosumab, and the non-licenced drug odanacatib. The analysis included 96,385 subjects, 6.8% of whom had T2D. The researchers examined the impact of treatment on fracture risk, BMD, and bone turnover markers in individuals with and without T2D. Overall, the results showed that antiresorptive treatments effectively reduced fracture risk and increased BMD, regardless of diabetes status (Fig. 2) [20]. The findings provide strong evidence that most licenced antiresorptive drugs effectively increase BMD and reduce fracture risk in individuals with T2D.

### Anabolic Drugs

Similarly to anti-resorptive drugs, there are no RCTs that assessed the effect of anabolic drugs in people with T2D. The current evidence comes from observational studies and post-hoc analyses of previous RCTs.

#### Teriparatide

Teriparatide is a recombinant form of parathyroid hormone. The daily administration leads to a transient increase in parathyroid hormone levels and anabolic effects on osteoblasts, stimulating bone formation and increasing BMD. Moreover, teriparatide enhances the differentiation of osteoblast precursors and promotes the incorporation of calcium into newly formed bone matrix. It is indicated for the treatment of severe osteoporosis in people with high fracture risk [27], as well as for individuals with osteoporosis associated with glucocorticoid use [28]. The pivotal trial has shown that teriparatide reduces the risk of vertebral and non-vertebral fractures [27], and a meta-analysis of observational studies has shown that it reduces the risk of hip fractures [29]. Common adverse reactions include dizziness, nausea, and leg cramps, typically occurring during the initial treatment phase. Additionally, teriparatide has been associated with an increased risk of hypercalcemia, particularly in patients with pre-existing hypercalcemia or conditions predisposing to hypercalcemia. Therefore, monitoring serum calcium levels is important during teriparatide therapy.

Another concern associated with teriparatide is the potential risk of osteosarcoma. Animal studies have shown an increased incidence of osteosarcoma in rats exposed to high doses of teriparatide. However, no conclusive evidence has established a similar risk in humans. As a precautionary measure, teriparatide is contraindicated in individuals with a history of skeletal malignancies or Paget’s disease of the bone. The use of teriparatide was initially limited to a maximum of 2 years. However, the Food and Drug Administration removed the boxed warning about the potential risk of osteosarcoma and the 2-year lifetime treatment limitation in the USA in 2020. A 15-year US post-marketing surveillance study found no evidence of increased osteosarcoma incidence associated with teriparatide use in adults. The observed number of osteosarcoma cases among teriparatide-treated patients was lower than expected based on the background incidence rate [30]. This study provides reassurance regarding the safety of teriparatide in relation to osteosarcoma risk. On stopping teriparatide, patients should be transitioned to antiresorptive medications for continued osteoporosis management.

**Fig. 3:**
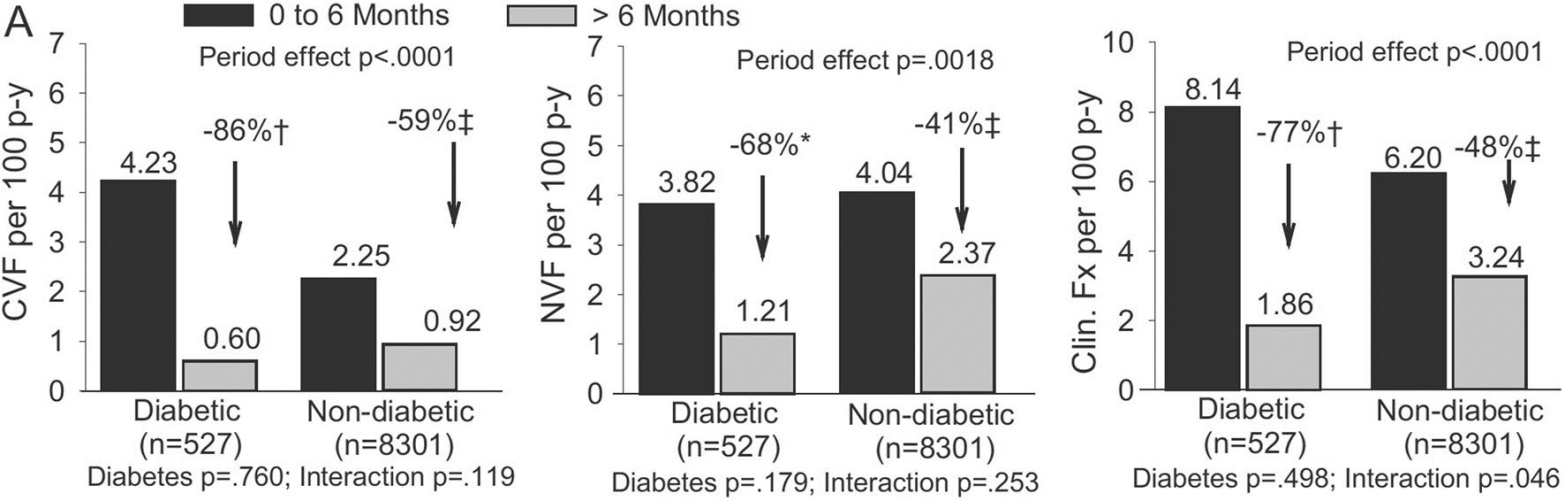
Fracture rates by diabetes mellitus presence use at baseline and treatment period. Shown are clinical vertebral fracture (left), non-vertebral (middle), and clinical fracture (right) rates per 100 patient-years for the reference period (0 to 6 months) versus post reference period (> 6 months) for subgroups based on diabetes mellitus presence at baseline. *p < 0.05; ^†^*p* < 0.005; ^‡^*p* < 0.0001 between periods. Time effect compares fracture rate between the 2 treatment periods irrespective of subgroup; interaction assesses whether time effect varied between subgroups; subgroup compares fracture rate between subgroups irrespective of period effect. Period and subgroup significant at *p* < 0.05; interaction significant at *p* < 0.10. Abbreviations: CVF, clinical vertebral fracture; Fx, fractures; NVF, non-vertebral fracture. Reproduced with permission from Langdahl et al. [32]

Current evidence suggests that the efficacy and safety of teriparatide are similar in patients with and without T2D. Real world observational data from 4042 patients, 291 of them with T2D, have shown teriparatide had similar effects on fracture incidence, BMD, and back pain in patients with and without T2D [31]. This study was included in another analysis that added three other observational studies [32]. The pooled analysis showed significant reductions in clinical vertebral fractures, non-vertebral fractures, clinical fractures, and hip fractures during teriparatide treatment in people with T2D compared to people without treatment. In this study, the reduction in clinical fractures was greater in people with T2D compared to people without T2D (Fig. 3). Therefore, the current evidence suggests that teriparatide presents similar efficacy and safety in people with and without T2D.

#### Abaloparatide

Abaloparatide is a synthetic analogue of parathyroid hormone-related protein (PTHrP) used to treat osteoporosis in postmenopausal women at high fracture risk. It acts as an anabolic agent, stimulating bone formation and improving BMD. Abaloparatide activates the PTH1 receptor, increasing osteoblastic activity and subsequent bone remodelling. Clinical trials have demonstrated its effectiveness in reducing the risk of vertebral and non-vertebral fractures. Common side effects of abaloparatide include hypercalcemia, nausea, and headaches. The data on the efficacy of abaloparatide in patients with T2D comes from the post-hoc analysis of the ACTIVE trial. In this trial, participants were randomised 1:1:1 to daily s.c. injections of placebo, abaloparatide (80 µg), or open-label teriparatide (20 µg) for 18 months. Abaloparatide led to significant (*p* < 0.001) improvements in BMD at the total hip, femoral neck, and lumbar spine, TBS at the lumbar spine, and fewer hip and vertebral fractures than placebo in the T2D group. There was a significant reduction in the risk of non-vertebral fractures [33]. These data suggest similar efficacy of abaloparatide in people with and without T2D.

### Drug with Dual Mechanisms of Action: Romosozumab

Romosozumab is a monoclonal antibody therapy that selectively inhibits sclerostin, a protein that normally suppresses bone formation, leading to increased osteoblastic activity and enhanced bone formation. Additionally, it transiently decreases the activity of osteoclasts, reducing bone resorption. This dual effect results in a rapid increase in BMD and reduced fracture risk. Clinical trials have demonstrated the efficacy of romosozumab in reducing the risk of vertebral, non-vertebral, and hip fractures [34].

Despite its promising effects, romosozumab is associated with certain side effects that require attention. Common adverse events include injection site reactions, arthralgia, and nasopharyngitis. Additionally, cardiovascular events have been reported at a slightly higher rate in the romosozumab-treated group compared to the group using BP [35]. This observation highlights the importance of carefully evaluating cardiovascular risk factors when considering romosozumab therapy for osteoporosis, and the treatment is not recommended for people with high cardiovascular risk.

Currently, there is no specific evidence on the efficacy or safety of romosozumab on people with T2D. T2D is associated with high sclerostin levels, making romosozumab an attractive alternative to treat this population [36]. However, there are concerns regarding the cardiovascular safety of romosozumab, and its use in patients with a high cardiovascular risk is not recommended (54). Experimental studies have not shown adverse effects of sclerostin inhibition on cardiovascular disease outcomes. Observational studies and clinical trials have yielded conflicting results, with some showing an increased risk of cardiovascular disease events with romosozumab treatment and others showing no effect [37]. Mendelian randomisation studies have used genetic variants that mimic the effects of sclerostin inhibition. Two out of three Mendelian randomisation studies provided further evidence for increased cardiovascular risk, with those genetically predisposed to lower sclerostin found to be at increased risk of myocardial infarction and T2D [38, 39] and one study finding greater coronary arterial calcification [38]. However, one Mendelian randomisation study did not find an effect of sclerostin lowering on the risk of myocardial infarction or T2D but found an effect on systolic blood pressure [40]*.* A pharmacovigilance study analysed serious cardiovascular events associated with romosozumab using data from the US FDA Adverse Event Reporting System (FAERS) from January 2019 to December 2020. The study found that romosozumab was associated with an increased risk of major cardiovascular events (such as myocardial infarction, stroke, and cardiac death) as well as other cardiovascular events (such as bleeding and thrombosis). These results were driven by the cases from Japan, where patients were older and more frequently male than reports from the USA [41]. Overall, the evidence suggests that sclerostin inhibition may increase the risk of cardiovascular disease, and further assessment of cardiovascular risk is needed when considering treatment with romosozumab. Given that T2D is associated with an increased risk of cardiovascular events, it is unlikely that the benefits of using romosozumab in this population would outweigh the risk of increasing cardiovascular risk.

## Conclusion

Osteoporosis and T2D are public health concerns. They impose significant burdens on patients, contribute to increased healthcare costs, and reduce quality of life, particularly for the elderly population. Despite no marked reduction in BMD and low bone turnover, individuals with T2D face an increased risk of fractures across various skeletal sites. These observations have raised concerns regarding the efficacy of anti-osteoporosis drugs in this population. Although no randomised controlled trials have specifically targeted individuals with T2D, observational studies and post-hoc analyses of RCTs have offered valuable insights into the effects of diverse anti-osteoporosis treatments in this patient group. Both anti-resorptive and anabolic agents have shown similar effectiveness in increasing BMD and reducing fractures in individuals with and without T2D. The current evidence suggests that most anti-osteoporosis drugs exhibit comparable efficacy in people with and without T2D. However, further investigations specifically designed for this population would be desirable.

## Data Availability

No datasets were generated during the current study. The review is based on the references cited.
